# Temperate Bacteriophages (Prophages) in *Pseudomonas aeruginosa* Isolates Belonging to the International Cystic Fibrosis Clone (CC274)

**DOI:** 10.3389/fmicb.2020.556706

**Published:** 2020-09-25

**Authors:** Antón Ambroa, Lucia Blasco, Carla López-Causapé, Rocio Trastoy, Laura Fernandez-García, Ines Bleriot, Manuel Ponce-Alonso, Olga Pacios, Maria López, Rafael Cantón, Timothy J. Kidd, German Bou, Antonio Oliver, Maria Tomás

**Affiliations:** ^1^Microbiology Department-Research Institute Biomedical A Coruña (INIBIC), Hospital A Coruña (CHUAC), University of A Coruña (UDC), A Coruña, Spain; ^2^Study Group on Mechanisms of Action and Resistance to Antimicrobials (GEMARA), Spanish Society of Infectious Diseases and Clinical Microbiology (SEIMC), Madrid, Spain; ^3^Microbiology Department-Health Research Institute of the Baleairc Islands (IdISBa), Hospital Son Espases, Palma de Mallorca, Spain; ^4^Servicio de Microbiología, Hospital Ramón y Cajal, Instituto Ramón y Cajal de Investigación Sanitaria (IRYCIS), Madrid, Spain; ^5^Spanish Network for Research in Infectious Diseases (REIPI), Seville, Spain; ^6^Child Health Research Centre, School of Chemistry and Molecular Biosciences, The University of Queensland, Brisbane, QLD, Australia

**Keywords:** prophages, inovirus, siphovirus, *Pseudomonas*, CC274 clone, cystic fibrosis

## Abstract

Bacteriophages are important in bacterial ecology and evolution. *Pseudomonas aeruginosa* is the most prevalent bacterial pathogen in chronic bronchopulmonary infection in cystic fibrosis (CF). In this study, we used bioinformatics, microbiological and microscopy techniques to analyze the bacteriophages present in 24 *P. aeruginosa* isolates belonging to the international CF clone (ST274-CC274). Interestingly, we detected the presence of five members of the *Inoviridae* family of prophages (Pf1, Pf4, Pf5, Pf6, Pf7), which have previously been observed in *P. aeruginosa*. In addition, we identified a new filamentous prophage, designated Pf8, in the *P. aeruginosa* AUS411.500 isolate belonging to the international CF clone. We detected only one prophage, never previously described, from the family *Siphoviridiae* (with 66 proteins and displaying homology with PHAGE_Pseudo_phi297_NC_016762). This prophage was isolated from the *P. aeruginosa* AUS531 isolate carrying a new gene which is implicated in the phage infection ability, named Bacteriophage Control Infection (*bci*). We characterized the role of the Bci protein in bacteriophage infection and in regulating the host Quorum Sensing (QS) system, motility and biofilm and pyocyanin production in the *P. aeruginosa* isogenic mutant AUS531Δ*bci* isolate. The findings may be relevant for the identification of targets in the development of new strategies to control *P. aeruginosa* infections, particularly in CF patients.

## Introduction

*Pseudomonas aeruginosa* is a ubiquitous Gram-negative microorganism and a multidrug-resistant (MDR) pathogen. It is the main pathogen that causes chronic respiratory infection in cystic fibrosis (CF) and is associated with substantial morbidity and mortality in CF patients.

Bacteriophages are bacterial viruses that infect bacteria. Phages generally undergo a lytic (virulent) or lysogenic (temperate) life cycle. Lytic phages enter host cells and subsequently lyse and kill them, releasing phage progeny into the surrounding medium. Temperate phages possess the ability to go through a lysogenic cycle, entering the host cell and integrating their nucleic acid in the host genome or residing in the host cells as prophages, potentially existing in a stable state for generations until induced to start a lytic cycle (Clokie et al., 2011).

Bacteriophages in the family *Inoviridae* (inoviruses) have been described in *P. aeruginosa* biofilms and as promoters of biofilm formation (Whiteley et al., 2001; Webb et al., 2004; Knezevic et al., 2015; Secor et al., 2015). Numerous studies have shown the relationship between CF clinical isolates and Pf filamentous prophages (Finnan et al., 2004; Kirov et al., 2007; Manos et al., 2008; Mathee et al., 2008; Winstanley et al., 2009; Fothergill et al., 2012), which are long, narrow, tubular phages (about 2 μm in length and 6–7 nm in diameter) with positive-sense single-stranded circular DNA (Secor et al., 2015). Pf phages are inoviruses and usually become integrated in the chromosome of *P. aeruginosa*, although there are some exceptions, such as Pf1, which can replicate without being integrated in the host strain (Secor et al., 2015).

The pathogenic potential of *P. aeruginosa* is probably due to a combination of many different virulence factors. Several studies suggest that these factors are regulated by Quorum Sensing (QS) systems and/or bacteriophages (Lee and Zhang, 2015). The QS network in this pathogen consists of a series of connected circuits, i.e., LasI/LasR, RhlI/RhlR, QscR and PqsABCDEH/PqsR, which are regulated by molecules known as acyl-homoserine lactones (Wilder et al., 2011; Lee and Zhang, 2015; Papenfort and Bassler, 2016). Detection of these molecules indicates that *P. aeruginosa* is growing as a biofilm within the lungs of CF patients (Bjarnsholt and Givskov, 2007; Wilder et al., 2009; Winstanley and Fothergill, 2009). This bacterium permanently colonizes the lungs of CF patients, despite antibiotic treatment being administered. Microscope studies of sputum samples from these patients show that *P. aeruginosa* frequently resides within biofilms (Bjarnsholt and Givskov, 2007). Specific detection of *P. aeruginosa* via QS signaling may help to identify the agents involved in biofilm formation.

Quorum sensing systems and bacteriophages are associated with virulence and evolution of bacteria during both intermittent and chronic lung infections in CF. Some studies have shown the existence of bacteriophages in the sputum of CF patients (Ojeniyi et al., 1991; Fothergill et al., 2011), supporting the hypothesis that the bacteriophages play a role in respiratory infections in these patients. A strain of *P. aeruginosa* known as the Liverpool epidemic strain (LES) shows greater resistance to antibiotics than other strains isolated from CF patients. Genomic analysis of isolate LESB58 has demonstrated the presence of several prophages that increase the success of colonization by this *P. aeruginosa* strain as they form part of the accessory genome, the genes of which contribute to pathogenicity (Winstanley et al., 2009).

Relationships between QS and bacteriophage infection have been analyzed by several authors. Phage φpa3 has been proved to transduce mutations in QS genes in P. aeruginosa PAO1 (Monson et al., 2011). Moreover, it was demonstrated that QS systems may protect bacteria from bacteriophage infection reducing the phage receptor numbers at the stationary phase in *Escherichia coli* (Tan et al., 2015). In *Vibrio cholerae*, QS was demonstrated to control the change from a lysogenic cycle to a lytic one in the vibrio phage VP882 by QS-related genes encoded by the bacteriophage itself 30554875 (Silpe and Bassler, 2019).

In the present study, 24 sequences of *P. aeruginosa* isolates belonging to the international CF clone (ST274-CC274) were analyzed. A new filamentous prophage, designated Pf8, was identified in isolate AUS411, and analysis of its genome revealed a toxin/antitoxin system. Moreover, a new prophage from the *Siphoviridae* family was identified in isolate AUS531, which harbors a new gene that favors phage infectivity and bacterial QS control, that was named Bacteriophage Control Infection (*bci*).

## Materials and Methods

### CF Clinical Isolates

All isolates (9 from CF Australian patients and 15 from Spanish patients from different clinical units), previously classified as belonging to CC274, were respiratory tract isolates from CF patients, except PAMB148, which was a blood sample. Isolates were recovered during an 18-year period (1995–2012) and included sequential isolates from several patients (López-Causapé et al., 2017). The antibiotic susceptibility profile and main antibiotic resistance-related mutations were previously analyzed (López-Causapé et al., 2017).

### Genome Sequencing and Analysis of the Isolates Belonging to the ST274 Clonal Complex (CC274)

Next Generation Sequencing (NGS) was performed in a previous study, with the MiSeq sequencing system (Illumina platform) (López-Causapé et al., 2017). The sequences were assembled using the Newbler Roche assembler and Velvet (Velvet v1.2.10^1^). Putative Open Reading Frames (ORFs) were predicted using the GeneMarkS gene prediction program (Lukashin and Borodovsky, 1998). The Blast2Go and RAST servers (Conesa et al., 2005; Aziz et al., 2008) were used for functional annotation of each predicted protein. Reconstructed phage sequences were analyzed using PHAST and PHASTER tools (Zhou et al., 2011; Arndt et al., 2016). All phage proteins detected were manually annotated using the Protein BLAST (Kent, 2002), HHpred tools (Söding et al., 2005), and InterProScan tools (Zdobnov and Apweiler, 2001) and were found to display ≥50% protein homology. Genome sequences of the AUS531phi phage and Pf8_ST274-AUS411 filamentous phage were constructed with the assistance of CSAR-web (Chen and Lu, 2018) and RAST (Aziz et al., 2008).

The presence of the *bci* gene in prophages throughout the NCBI Nucleotide sequence was checked using BLAST, and its presence in a prophage was confirmed by PHASTER analysis of the bacterial genome. Protein domains of the protein were searched with CD-search in BLAST. Promoter regions were predicted with BPROM tool of SoftBerry^2^.

### Isolation of Clinical Temperate Phages From the ST274 Clonal Complex (CC274)

An overnight culture of the clinical *P. aeruginosa* isolate AUS531 was diluted in Luria-Bertani (LB) medium and grown for 2.5 h until reaching an OD_600_ (optical density measured at a wavelength of 600 nm) of 0.6, before being treated with mitomycin C (MMC). MMC was added at a concentration of 10 μg/ml and the culture was incubated at 37°C and shaken at 180 rpm until the cells were lysed. The lysate was incubated in the presence of chloroform for 20 min and centrifuged at 3400 × *g* for 10 min. Finally, the supernatant was filtered through a 0.45 nm filter (Millipore).

### Transmission Electron Microscopy (TEM) Examination of Temperate Phages: Inoviruses and Siphoviruses

Concentrated phage preparations were required for transmission electron microscopy (TEM). Phage particles were precipitated overnight at 4°C with polyethylene glycol (PEG6000) 3∼5% (w/v) and 0.5 M NaCl. The solution was centrifuged at 11000 × *g* at 4°C for 15 min. The pellet was resuspended with SM buffer (100 mM NaCl; 8 mM MgSO_4_ 7H_2_O; 50 mM Tris–HCl pH 7.5) and stored at 4°C. Samples were negatively stained with 1% aqueous uranyl acetate before examination by electron microscopy (Hargreaves et al., 2013).

### Characterization of Siphovirus Temperate Phage in Relation to Quorum Sensing

#### *bci* Deleted Strain and Phage Isolation

To obtain a strain without the *bci* gene for experiments and to subsequently obtain the AUS531phiΔ*bci* mutant phage, the *bci* gene was amplified with 1 kb upstream and downstream regions for deletion in the *P. aeruginosa* AUS531 isolate. The fragment was cloned into the pEX18Gm vector (GenBank: AF047518.1) (Hoang et al., 1998) using the UP_bci(*Kpn*I)/UP_Bci(*Xho*I) combination of primers for the upstream region and the DOWN_Bci(*Xho*I)/DOWN_Bci(*Bam*HI) combination for the downstream region (Table 1). Fragments were digested with *Kpn*I and *Xho*I restriction enzymes (upstream fragment) and *Xho*I and *Bam*HI (downstream region). Products were ligated into the pEX18Gm plasmid, and the recombinant plasmid was transformed in *E. coli* TG1 by electroporation.

**TABLE 1:** Primers and probes used in this study.

**PCR *P. aeruginosa* AUS531 mutant (AUS531Δ*bci*)**			
**Primer**	**Sequence (5′-3′)**	**Restriction Site**	**Reference**

UP_Bci Fow	GGGGGTACCGCACCGCAACCTCCCGATCA	*Kpn*I	This study
UP_Bci Rev	GGGCTCGAGGGCGTAACTCCGTTCGAGGG	*Xho*I	This study
DOWN_Bci Fow	GGGCTCGAGCGCCTGGCCTATTGCCGGGC	*Xho*I	This study
DOWN_Bci Rev	GGGGGATCCGTCGTCGATGATTGAGCGAA	*Bam*HI	This study
INT UP Fow	ATTGTAGTCATACTCAAGAC	–	This study
INT DOWN Rev	TGCACCGCCTTATGTGAAAG	–	This study
pEX18 Fow	GGCTCGTATGTTGTGTGGAATTGTG	–	This study
pEX18 Rev	GGATGTGCTGCAAGGCGATTAAG	–	This study
**RT-qPCR *P. aeruginosa* AUS531 mutant (AUS531Δ*bci*)**			
**Primer**	**Sequence (5′-3′)**	**UPL probe^*a*^**	**References**

proC_149_Fw	CTGTCCAGCGAGGTCGAG	149	Tan et al., 2015
proC_149_Rev	CCTGCTCCACCAGTGCTT		
LasR_139_Fw	GATATCGGTTATCTGCAACTGCT	139	This study
LasR_139_Rev	CCGCCGAATATTTCCCATA		
RhlR_115_Fw	TGCGTTGCATGATCGAGT	115	This study
RhlR_115_Rev	CGGGTTGGACATCAGCAT		
QscR_133_Fw	GTTCCAGCGAGAGCATCG	133	This study
QscR_133_Rev	TGGTGATCCAGAGCAGGAA		
PqsR_151_Fw	TCGACACCAAGGTGTATTGC	151	This study
PqsR_151_Rev	TCGAGAAAGCGCAGGAAG		

*^*a*^Universal Probe Library (UPL) (Roche, Germany; https://lifescience.roche.com/en_es/brands/universal-probe-library.html).*

The resulting plasmid was used to transform the *P. aeruginosa* AUS531 isolate by electroporation for genomic recombination and resulting gene knockout. Recombinant colonies representing the first crossover event were obtained by gentamicin-mediated selection. Gentamicin-resistant colonies were grown overnight in LB supplemented with 15% sucrose, and they were then plated on the same medium. Secondary crossover events were confirmed by PCR and by sequencing with the primers listed in Table 1. The AUS531phiΔ*bci* phage was obtained from the mutant AUS531Δ*bci* strain by induction with MMC, as previously described.

#### Expression of the *bci* Gene in Relation to QS Genes by RT-PCR

To establish the relationship between *bci* gene and QS, we measured the *bci* gene expression in the AUS531 strain incubated in the presence of QS signals. One colony of each of *P. aeruginosa* isolates AUS531 and AUS531Δ*bci* was inoculated in LB broth and incubated overnight at 37°C under stirring at 180 rpm. The overnight culture was diluted (1:100) and allowed to grow until reaching an OD_600_ of 0.3. Aliquots of 10 μL of QS-system signals 3-Oxo-C12-HSL (Stacy et al., 2012; López et al., 2017) and N-butanoyl-L-HSL (C4-HSL, which regulates through QS) and the same volume of DMSO as used in controls were added. The samples were incubated for 1 h (Karig and Weiss, 2005; Dubeau et al., 2009; Zhang et al., 2013). RNA was extracted using the High Pure RNA Isolation kit (Roche, Germany), and the extract was treated with DNAse (Roche, Germany). The extracted RNA measured was in a NanoDrop ND-100 spectrophotometer (NanoDrop Technologies). The concentration of the samples was adjusted to 50 ng/μL to yield efficiencies of 90–110% (Rumbo et al., 2013). The expression studies were carried out with Lightcycler 480 RNA Master Hydrolysis Probe (Roche, Germany), under the following conditions: reverse transcription at 63°C for 3 min, denaturation at 95°C for 30 s, followed by 45 cycles of 15 s at 95°C and 45 s at 60°C and, finally, cooling at 40°C for 30 s. In all of the experiments, the final volume was 20 μL per well (18 μL of master mix and 2 μL of RNA at 50 ng/μL). Primers and the respective Universal Probe Library (UPL) probes are listed in Table 1. For each isolate, expression of all genes, primers and probes was normalized relative to the reference or housekeeping gene, *proC* (Savli et al., 2003). All of the samples were analyzed in triplicate. Statistically significant differences were determined by Student’s *t*-test (GraphPad Prism v.6).

In order to analyze the effect of the phage with and without *bci* gene on QS, we analyzed the expression of QS genes (*lasR*, *rhlR*, *qscR*, and *pqsR*) in AUS531Δ*bci* incubated for 30 min with AUS531phi and AUS531phiΔ*bci* phages in an early step of bacterial growth. An overnight culture was diluted (1:100) in LB broth with 10 mM MgSO4 and 10 mM CaCl_2_ and then grown until reaching an OD_600_ of 0.2–0.4. Both wild type AUS531phi and AUS531phiΔ*bci* phages were added at a multiplicity of infection (MOI) of 10. All controls were prepared by adding the same volume of phage buffer. RNA extraction and expression studies were carried out in the same way as in the previous step. All of the samples were analyzed in triplicate. Statistically significant differences were determined by Student’s *t*-test.

#### Effect of the *bci* Gene Interaction Carried by Bacteriophage on the QS: Infective Capacity, Biofilm Production, Bacterial Motility and Pyocyanin Secretion

To characterize the infection curve for the bacteriophages, an overnight culture of *P. aeruginosa* AUS531Δ*bci* was diluted 1:100 in LB broth supplemented with MgSO_4_ and CaCl_2_ (both at a concentration of 10 mM). The mixture was incubated at 37°C at 180 rpm until reaching an OD_600 nm_ of 0.1, before being infected with the phage AUS531phi and with the phage AUS531phiΔ*bci* at a MOI of 1 and 10. Measurements were made during 6 h at 1-h intervals. Statistically significant differences were determined by Student’s *t*-test (GraphPad Prism v.6) by comparing the data obtained every hour.

To study the effect on bacterial motility, an overnight culture of *P. aeruginosa* AUS531Δ*bci* was diluted 1:100 in LB broth with 10 mM MgSO_4_ and 10 mM CaCl_2_ until reaching an OD_600_ of 0.5∼0.6. A spot of 1 μL of a mixture of AUS531Δbci culture and each phage (wild type AUS531phi and mutant AUS531phiΔbci at a MOI of 1) was placed in plates containing LB medium and 0.3% agar supplemented with 10 mM MgSO_4_ and 10 mM CaCl_2_ (Clemmer et al., 2011).

To study the effect on biofilm production, we used the modified version of the biofilm formation assay (O’Toole, 2011). Briefly, an overnight culture of *P. aeruginosa* AUS531Δ*bci* was adjusted to 10^7^ CFU/mL in LB broth supplemented with 10 mM MgSO_4_, 10 mM CaCl_2_ and 2% glucose, and 100 μL was finally added to each well of a “U”-bottom 96-well microtiter plate and incubated at 37°C for 24 h. Thirty wells were infected at MOI 10 with AUS531phi wild temperate phage and the other 30 with AUS531Δ*bci* mutant strain. Planktonic cell growth was measured at OD_600_ before being removed. The cells were rinsed three times with distilled water and then fixed at 60° for 1 h. Biofilms were stained with 125 μL of 0.4% crystal violet (CV) for 15 min, washed four times with distilled water, and the CV retained was solubilized with 125 μL of 30% acetic acid and measured at OD_595_. Biofilm production was calculated by dividing the OD_595_ of the CV-stained culture by the OD_600_ of the growth for each well. Statistical differences were determined with a Student’s *t*-test. In order to confirm integration of the temperate phages, the presence of the *bci* gene was checked by PCR in 10 isolated colonies in each biofilm assay.

To analyze pyocyanin secretion, an overnight culture of *P. aeruginosa* AUS531Δ*bci* was diluted 1:100 in 10 mL of LB broth enriched with 10 mM MgSO_4_ and 10 mM CaCl_2_ and then grown until an OD_600_ of 0.2 was reached. The culture was then infected with phages AUS531phi and AUS531phiΔ*bci* at 10 MOI and incubated for 6 h. The pyocyanin was extracted by adding 6 mL of chloroform to the culture and incubating the solution for 2 h at 37°C under continuous stirring at 180 rpm. Two mL of 0.2 N HCl was then added to yield a pink to deep red solution. The absorbance of this solution was measured at an OD of 520 nm. The concentrations, expressed as micrograms of pyocyanin produced per milliliter of culture supernatant (μg/mL), were determined by multiplying the optical density at 520 nm by 17.072 (Essar et al., 1990; Clemmer et al., 2011). Statistically significant differences were determined by Student’s *t*-test (GraphPad Prism v.6).

## Results

### Analysis of QS Network and Temperate Phages in the Genome of *P. aeruginosa* CF Clone (ST274-CC274) Isolates

**FIGURE 1:**
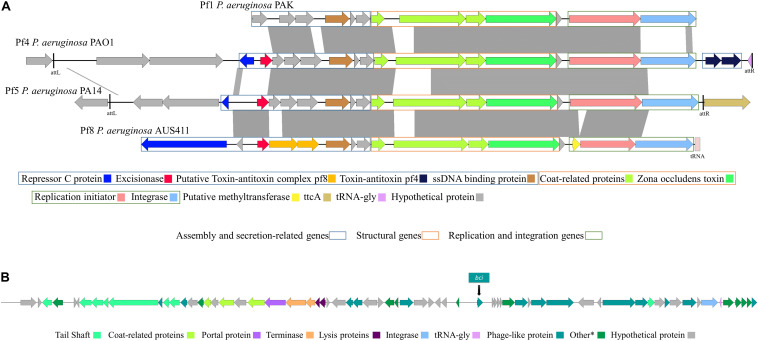
**(A)** Schematic representation and comparison of filamentous phages Pf1, Pf4, Pf5, and Pf8. Genes are classified by function into assembly and secretion, structural, and replication/integration genes. Dark gray regions represent >90% of nucleotide sequence identity between Pf genome regions. **(B)** Schematic representation of the genome of siphovirus phage AUS531phi and position of the Bci protein (GenBank: MN585195.1).

We performed a genomic analysis of the 24 *P. aeruginosa* isolates belonging to the ST274 clonal complex (CC274) obtained from CF patients and of the reference *P. aeruginosa* PAO1 strain genome (GenBank: AE004091.2) (Table 2). Complete prophage sequences were present in three strains in the *P. aeruginosa* sequences: AUS411, AUS531, and FQSE15-1110 (Table 2). Three of these showed high similarity to the *Pseudomonas* Pf inovirus, constituted by 9-15 proteins in isolates AUS411, AUS531, and FQSE15-1110. The inoviruses present in isolates AUS531 and FQSE15 were similar to the *Pseudomonas* Pf4 and Pf5 inoviruses, but the prophage detected in AUS411 was a new phage, designated Pf8_ST274-AUS411 (hereinafter referred to as Pf8) (Hay and Lithgow, 2019; Li et al., 2019). The genome of the Pf8 filamentous phage is of size 10 Kb and has a total of 16 proteins and one tRNA coding region (Genbank:MN710383). It has a GC content of 58.1%. Interestingly, Pf8 showed high protein identity with the filamentous bacteriophages Pf4 (*P. aeruginosa* PAO1) and Pf5 (*P. aeruginosa* PA14) (Mooij et al., 2007) (Figure 1A). However, new proteins involved in viral defense were identified in the Pf8 bacteriophage, including a putative toxin-antitoxin module (Genbank: QGZ15329.1 and QGZ15330.1) and methyltransferase (Genbank: QGZ15339.1). The prophage designated AUS531phi (accession number MN585195), detected in isolate AUS531 was found to be homologous with the *Pseudomonas* Phi297 bacteriophage.

**TABLE 2:** Cystic Fibrosis clone isolates in the study (ST274-CC274) and their complete prophage presence.

Isolate	Location	Year	Prophage	KB	ORF	Homology (PHASTER)
AUS034	Australia	2008	0	–	–	
AUS037	Australia	2008	0	–	–	
AUS410×	Australia	2007	0	–	–	
AUS411	Australia	2007	1	5.5	9	PHAGE_ Pseudo_ Pf1_ NC_ 001331(9)
AUS531	Australia	2008	1	48	62	PHAGE_ Pseudo_ phi297_ NC_ 016762(22)
			2	4.7	10	PHAGE_ Pseudo_ Pf1_ NC_ 001331(9)
AUS588×	Australia	2008	0	–	–	
AUS601	Australia	2008	0	–	–	
AUS603	Australia	2008	0	–	–	
AUS690	Australia	2008	0	–	–	
FQRC10	Spain	1995	0	–	–	
FQRC15	Spain	1997	0	–	–	
FQRC26	Spain	1995	0	–	–	
FQSE03-1212	Spain	2012	0	–	–	
FQSE06-0403	Spain	2003	0	–	–	
FQSE06-0610	Spain	2010	0	–	–	
FQSE10-0110	Spain	2010	0	–	–	
FQSE10-0111	Spain	2011	0	–	–	
FQSE10-0503	Spain	2003	0	–	–	
FQSE15-0803	Spain	2003	0	–	–	
FQSE15-0906	Spain	2006	0	–	–	
FQSE15-1110	Spain	2010	1	7.9	15	PHAGE_ Pseudo_ Pf1_ NC_ 001331(9)
FQSE24-0304	Spain	2004	0	–	–	
FQSE24-1010	Spain	2010	0	–	–	
PAMB148	Spain	2010	0	–	–	

The genome of the AUS531phi prophage is almost 50 Kb in size and contains a total of 66 proteins, one tRNA coding region and 63% GC content. The genome of the AUS531phi (Figure 1B) carries prophage assembly proteins, such as tail shaft proteins (GenBank: QGF21321.1, QGF21325.1, QGF21326.1, QGF21327.1, QGF21328.1, QGF21330.1, QGF21331.1, and QGF21373.1), coat-related proteins (GenBank: QGF21339.1, QGF21337.1, and QGF21335.1), a portal protein (GenBank: QGF21340.1), terminase proteins (GenBank: QGF21341.1 and QGF21342.1), lysis proteins (GenBank:QGF21343.1 and QGF21344.1), an integrase (GenBank: QGF21379.1) and other phage-related proteins. The genome harbors a carbon storage regulator (Genbank: QGF21359.1) (QS regulator associated with biofilm inhibition), called Bci protein (Figure 1B). The *bci* gene has 372 bp and the Bci protein has 123 amino acids (Supplementary Figures S1A,B, respectively) with a promoter region in the upstream sequence between the nucleotides 30327 and 30372 (Figure S1C) (GenBank: MN585195). There is a putative *rhl*-*las* box with a motif CT-(N13)-AG between the nucleotides 30342 and 30358 (Figure S1C and Supplementary Material). Following a CD-search in BLAST, CsrA superfamily domain is present between amino acids 1 and 51 with an *e*-value of 2.77e-27. We analyzed the distribution of the *bci* gene among the *P. aeruginosa* genomes deposited in the NCBI database (Table 3). We found that this gene was present in 33 different *P. aeruginosa* strains, with high homology (>95% of protein homology in most of these sequences). Furthermore, we found (using the PHASTER search tool) that the *bci* gene was only present in prophage sequences in these strains (Table 3). Moreover, in 6 of the isolates, the DNA region in this gene showed high homology (>95%) with repeat sequences of previously characterized systems (Table 3), showing that the *bci* gene present in phages as CRISPR sequences are constructed with previously infected DNA bacteriophage fragments (Cady et al., 2011).

**TABLE 3:** Genomes of the *P. aeruginosa* isolates carrying bacteriophages with the *bci* gene and homologous CRISPR system in the *bci* gene.

Genome of *Pseudomonas* harboring bacteriophage with *bci* gene	Sequence ID	BLAST Homology (%)	Homologous Phage identified by PHASTER tool
*Pseudomonas aeruginosa* strain Ocean-1175	CP022525.1	99	PHAGE_Pseudo_phi297_NC_016762(25)
*Pseudomonas* sp. AK6U	CP025229.1	98	PHAGE_Pseudo_phi297_NC_016762(39)
*Pseudomonas aeruginosa* strain Pa1242	CP022002.1	98	PHAGE_Pseudo_phi297_NC_016762(21)
*Pseudomonas aeruginosa* strain E6130952	CP020603.1	98	PHAGE_Pseudo_phi297_NC_016762(39)
*Pseudomonas aeruginosa* strain N17-1	CP014948.1	97	PHAGE_Pseudo_YMC11/07/P54_PAE_BP_NC_030909(12)
*Pseudomonas aeruginosa* strain 97	CP031449.1	97	PHAGE_Pseudo_YMC11/02/R656_NC_028657(24)
*Pseudomonas aeruginosa* M18	CP002496.1	97	PHAGE_Pseudo_phi297_NC_016762
*Pseudomonas aeruginosa* strain AR_458	CP030327.1	97	PHAGE_Pseudo_phi297_NC_016762(23)
		95	PHAGE_Pseudo_phi297_NC_016762(15)
*Pseudomonas aeruginosa* strain AR439	CP029097.1	97	PHAGE_Gordon_Schwabeltier_NC_031255(62)
*Pseudomonas aeruginosa* strain M28A1	CP015649.1	97	PHAGE_Pseudo_YMC11/02/R656_NC_028657(20)
*Pseudomonas aeruginosa* strain F63912	CP008858.2	97	PHAGE_Pseudo_phi297_NC_016762(11)
*Pseudomonas aeruginosa* strain H5708	CP008859.2	97	PHAGE_Pseudo_YMC11/02/R656_NC_028657(26)
*Pseudomonas aeruginosa* RP73	CP006245.1	97	PHAGE_Pseudo_phi297_NC_016762(11)
*Pseudomonas aeruginosa* strain CCUG 70744	CP023255.1	96	PHAGE_Pseudo_phi297_NC_016762(29)
*Pseudomonas aeruginosa* strain PPF-1	CP023316.1	96	PHAGE_Gordon_Schwabeltier_NC_031255(65)
*Pseudomonas aeruginosa* strain F30658	CP008857.1	96	PHAGE_Pseudo_YMC11/02/R656_NC_028657(23)
*Pseudomonas aeruginosa* strain PB368	CP025050.1	96	PHAGE_Pseudo_phi297_NC_016762(20)
*Pseudomonas aeruginosa* strain PB369	CP025049.1	96	PHAGE_Pseudo_phi297_NC_016762(20)
*Pseudomonas aeruginosa* strain PA_150577	CP017306.1	96	PHAGE_Pseudo_YMC11/02/R656_NC_028657(27)
*Pseudomonas aeruginosa* strain PA121617	CP016214.1	96	PHAGE_Pseudo_YMC11/02/R656_NC_028657(20)
*Pseudomonas aeruginosa* strain W16407	CP008869.2	95	PHAGE_Pseudo_phi297_NC_016762(43)
*Pseudomonas aeruginosa* strain AR442	CP029090.1	95	PHAGE_Pseudo_phi297_NC_016762(42)
*Pseudomonas aeruginosa* strain T63266	CP008868.1	95	PHAGE_Pseudo_phi297_NC_016762(16)
*Pseudomonas aeruginosa* strain ATCC 27853	CP015117.1	96	PHAGE_Pseudo_phi297_NC_016762(37)
*Pseudomonas aeruginosa* DNA, complete genome, strain: 8380	AP014839.2	96	PHAGE_Pseudo_phi297_NC_016762(44)
*Pseudomonas aeruginosa* strain CCBH4851	CP021380.1	94	PHAGE_Pseudo_JBD44_NC_030929(31)
*Pseudomonas aeruginosa* strain PA7790	CP014999.1	94	PHAGE_Pseudo_phi297_NC_016762(22)
*Pseudomonas aeruginosa* strain PA8281	CP015002.1	94	PHAGE_Pseudo_phi297_NC_016762(22)
*Pseudomonas aeruginosa* strain AR_0446	CP029660.1	94	PHAGE_Pseudo_YMC11/07/P54_PAE_BP_NC_030909(25)
*Pseudomonas aeruginosa* PA7	CP000744.1	93	PHAGE_Pseudo_phi297_NC_016762(24)
*Pseudomonas aeruginosa* strain PASGNDM699	CP020704.1	92	PHAGE_Pseudo_YMC11/02/R656_NC_028657(23)
*Pseudomonas aeruginosa* strain PASGNDM345	CP020703.1	92	PHAGE_Pseudo_YMC11/02/R656_NC_028657(23)
*Pseudomonas aeruginosa* strain BAMCPA07-48	CP015377.1	89	PHAGE_Pseudo_YMC11/02/R656_NC_028657(27)

**Strains with homologous CRISPR system in the *bci* gene**	**Sequence ID**	**BLAST Homology (%)**	

*Pseudomonas aeruginosa* strain SMC4395 CRISPR repeat sequence	HQ326191.1	100	
*Pseudomonas aeruginosa* strain SMC4498 CRISPR repeat sequence	HQ326189.1	97	
*Pseudomonas aeruginosa* strain SMC4494 CRISPR repeat sequence	HQ326188.1	97	
*Pseudomonas aeruginosa* strain SMC4489 CRISPR repeat sequence	HQ326187.1	97	
*Pseudomonas aeruginosa* strain F63912	CP008858.2	97	
*Pseudomonas aeruginosa* RP73	CP006245.1	97	

*(https://www.ncbi.nlm.nih.gov/genome/browse/#!/overview/).*

### TEM Micrographs of Bacteriophages

**FIGURE 2:**
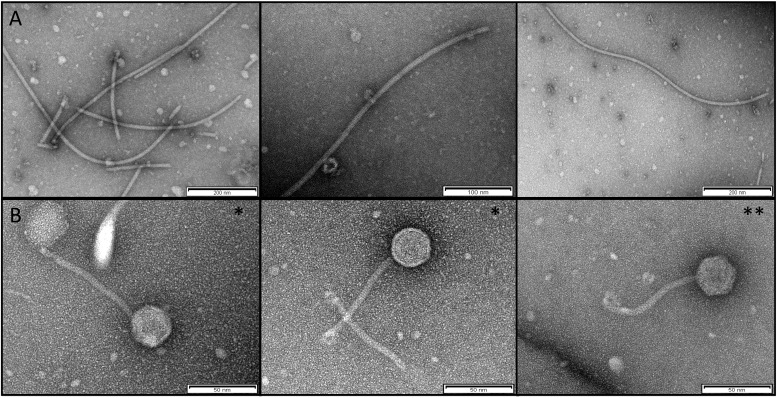
**(A)** TEM of the *Inoviridae* Pf8 bacteriophage of *P. aeruginosa* clinical isolate AUS411 (Pf8_ST274-AUS411). **(B)** TEM of *Siphoviridae* bacteriophage of *P. aeruginosa* clinical isolates AUS531 (AUS531phi*) and AUS531Δ*bci* (AUS531Δ*bci*phi**).

We confirmed the presence of *Inoviridae* type phages by TEM examination of extracts of overnight supernatant cultures of *P. aeruginosa* isolate AUS411 (Figure 2A). Moreover, we confirmed the presence of the *Siphoviridae* type phages AUS531phi and AUS531phiΔ*bci* by TEM examination of the preparations (Figure 2B). The morphology of the structures seen in the pictures is clearly that of *Siphoviridae* type phages (Alič et al., 2017).

### Relationship Between the Bacteriophages and the QS System

#### Gene Expression

**FIGURE 3:**
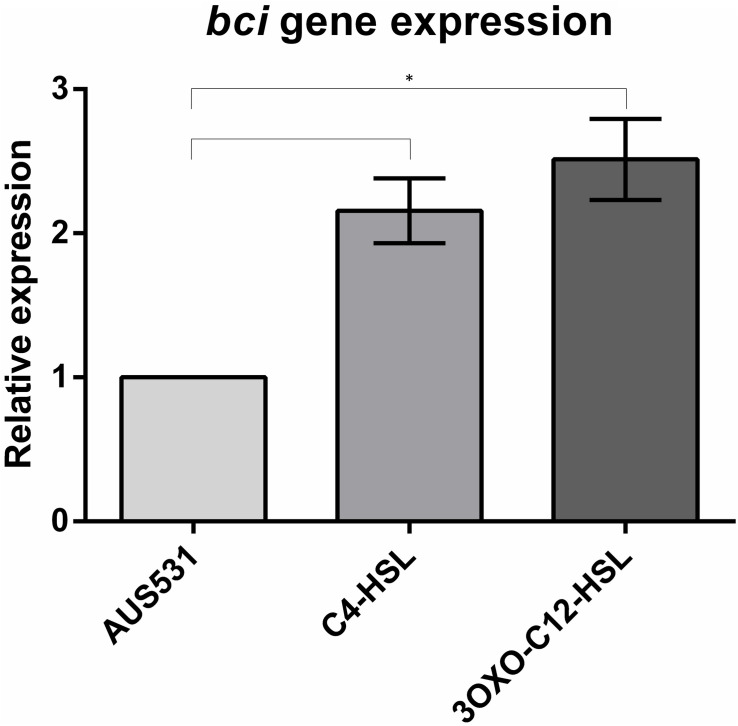
Relative expression of the *bci* gene in AUS531 isolate under the quorum sensing inducers C4-HSL and 3OXO-C12-HSL. Standard deviations are indicated. (^∗^) Statistically significant differences (*p* < 0.05) were determined by Student’s *t*-test (GraphPad Prism v.6).

We observed an increase in the expression of the *bci* gene in the prophage region in the presence of two acyl-homoserine lactone QS inducers: 3-oxo-C12-HSL and C4-HSL (Figure 3), demonstrating that the *bci* gene is associated with the QS system.

**FIGURE 4:**
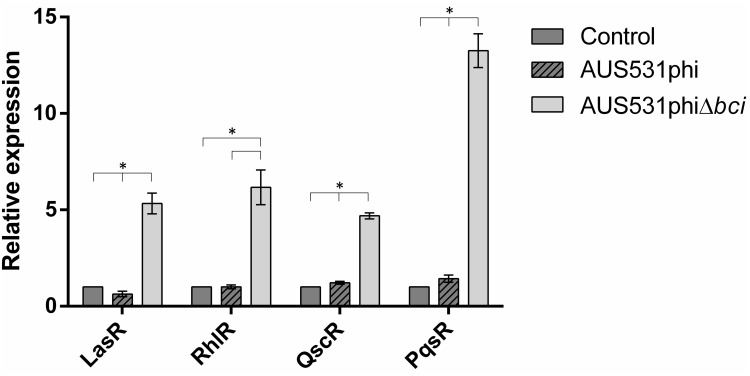
Relative expression of quorum sensing-related genes of the AUS531Δ*bci* isolate infected with AUS531phi and AUS531phiΔ*bci* bacteriophages. Standard deviations are indicated. (^∗^) Statistically significant differences (*p* < 0.05) were determined by Student’s *t*-test (GraphPad Prism v.6).

In addition, we studied the effect of infection by bacteriophages AUS531phi and AUS531phiΔ*bci* in the expression of the genes *lasR*, *rhlR*, *qscR*, and *pqsR* of the QS in AUS531Δ*bci* bacterial strain to check the effect of the gene in the first step of bacteriophage infection (Figure 4). The graphic representation shows a fold change of around 5.0 for *lasR*, *lhlR*, and *qscR* when isolate AUS531Δ*bci* was infected with the mutant phage AUS531phiΔ*bci*, while infection with the wild phage AUS531phi yielded fold changes of around 1.0 in these genes. The differences in the expression for infection with a phage containing a *bci* gene and in the absence of this gene suggest that these genes are involved in regulating the QS system in order to overcome it and infect the bacteria. Interestingly, there was a fold change of around 13.0 in the *pqsR* gene when isolate AUS531Δ*bci* was infected with phage AUS531phiΔ*bci*, in contrast to a fold change of around 1.5 when the isolate was infected with the wild phage AUS531phi.

#### Infection Curve

**FIGURE 5:**
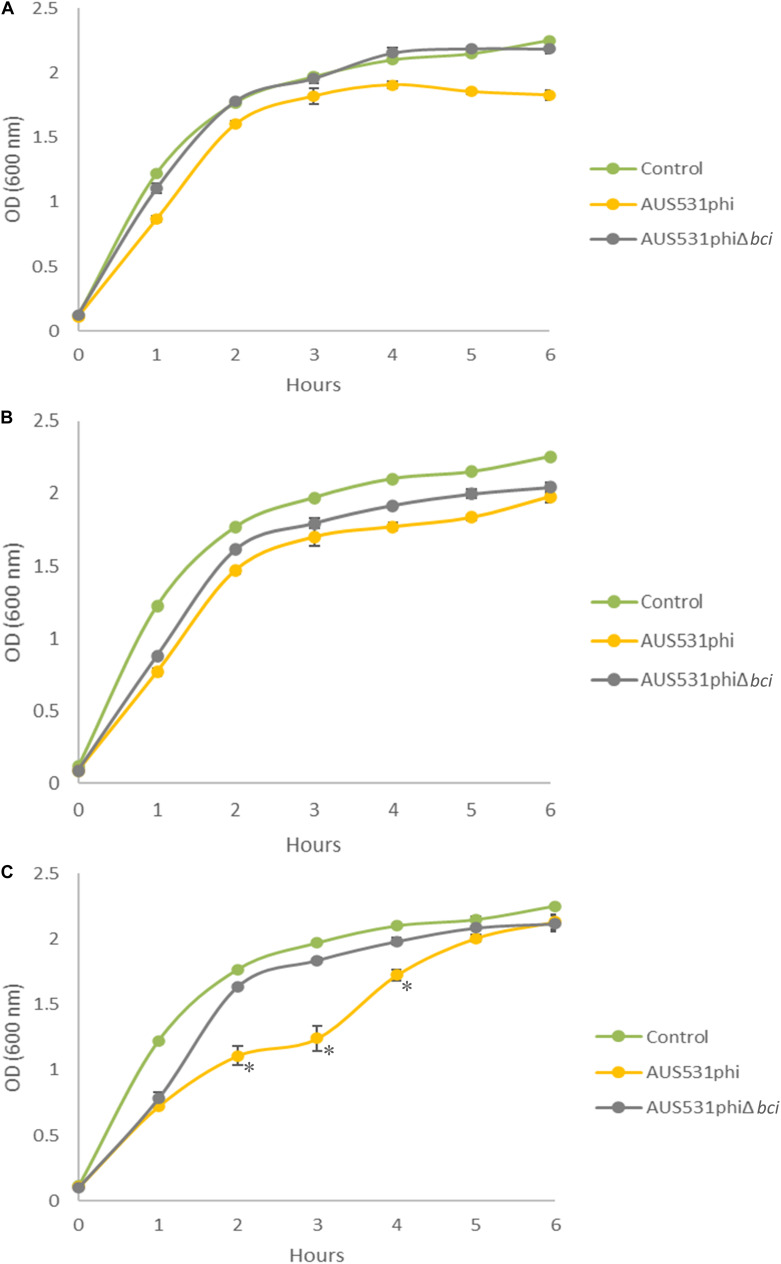
Infection curves for the lysogenic phages AUS531phi **(A)** and AUS531phiΔ*bci* at MOIs 0.1 **(A)**, 1 **(B)**, and 10 **(C)** during 6 h. Standard deviations are indicated. Statistically significant differences were determined by Student’s *t*-test for each point on the curve (GraphPad Prism v.6). (*) indicates a strongly significant difference (*p* < 0.0001).

The relationship between the *bci* gene and the ability of the phage to infect the host was demonstrated in the infection curves. The host isolate AUS531Δ*bci* grew less when infected with the wild type phage AUS531phi than when it was infected with the mutated phage, AUS531phiΔ*bci* at all the MOI assayed (Figure 5). The infection curves for phage AUS531phi were significantly different (*p* < 0.05) at MOI 0.1, 1 and also at MOI 10 (*p* < 0.0001). In addition, the infection curves for phage AUS531phi were significantly different from the corresponding control curves at MOI 1 and 10 (*p* < 0.05), but not at MOI 0.1. Comparison of the growth of the culture infected with the wild type phage AUS531phi and the mutant phage AUS531phiΔ*bci* revealed significant differences at MOI 0.1 and 1 (*p* < 0.05) (Figures 5A,B) at all time points measured, and at MOI10 the differences were significant (*p* < 0.0001) (Figure 5C) at 2, 3, and 4 h. These results indicate that capacity of infection of AUS531phi is higher than that of AUS531Δ*bci*, thus confirming that the *bci* gene is related to the infection capacity of this phage.

#### Relationship Between the Phage Infection and Virulence Factors: Motility, Biofilm and Pyocyanin Production

**FIGURE 6:**
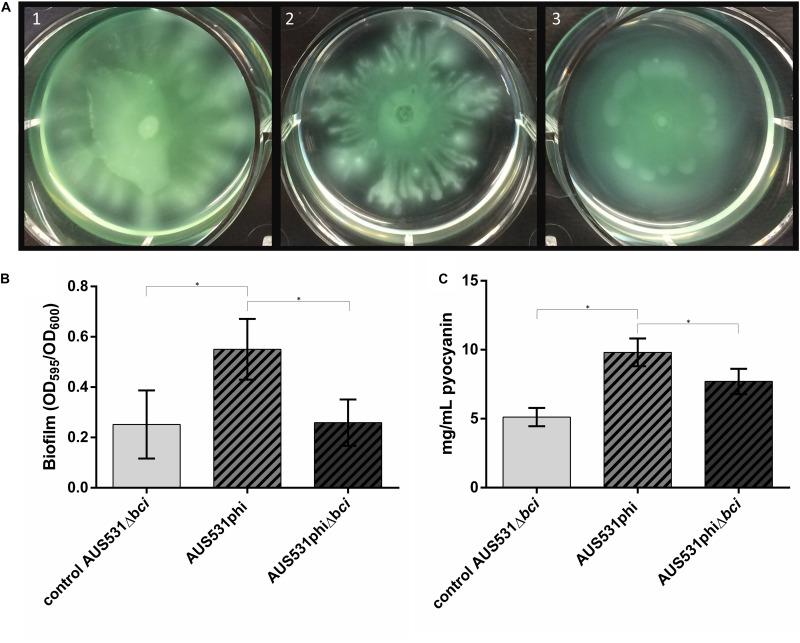
**(A)** Motility assay in AUS531Δ*bci* under normal conditions (1), adding AUS531phi phage (2), and adding AUS531phiΔ*bci* phage (3). **(B)** Biofilm production after 24 h of AUS531 wild type isolate, AUS531Δ*bci* under normal conditions, AUS531Δ*bci* in response to addition of the wild type AUS531phi phage and the mutant AUS531Δ*bci* phage. (^∗^) Statistically significant differences were determined by Student’s *t*-test (GraphPad Prism v.6). **(C)** Pyocyanin production of controls AUS531 and AUS531Δ*bci* and AUS531Δ*bci* in response to infection with the wild-type strain.

In order to verify the relationship between the *bci* gene from the bacteriophage and bacterial virulence, we performed motility, biofilm and pyocyanin assays. When isolate AUS531Δ*bci* was infected with the wild type phage AUS531phi, a reduction in motility was observed. By contrast, when the same isolate was infected with the *bci* gene deleted from prophage AUS531phiΔ*bci*, there was no difference in motility relative to the control (Figure 6A). When isolate AUS531Δ*bci* was infected with the wild type phage AUS531phi, enhanced biofilm production was observed relative to the infection of mutant phage AUS531Δ*bci* (Figure 6B). The PCR of the biofilm isolated colonies showed the presence of the *bci* gene in strain AUS531Δ*bci* infected with the wild-type phage, thus confirming integration of this phage in the genome. Similarly, pyocyanin secretion was higher with the AUS531phi bacteriophage than with the AUS531phiΔ*bci* bacteriophage (Figure 6C) confirming that the *bci* gene influences bacterial virulence.

## Discussion

Cystic fibrosis is the main life-limiting recessive genetic disorder in the Caucasian population. It affects multiple organs, but is particularly damaging to the lungs. Colonization of the respiratory tract by some pathogens such as *P. aeruginosa* exacerbates the severity of the disease in CF patients (Rey et al., 2018).

Temperate bacteriophages of *P. aeruginosa* are involved in the horizontal transfer of DNA and show selective preference for developing and accumulating in the specific conditions of the lower lung (Tariq et al., 2015). Although most phages are pathogens that kill their bacterial hosts, filamentous phages live together with their host (Mai-Prochnow et al., 2015). Filamentous phages are widely distributed in Gram-negative bacteria and they have a strong impact on the physiology, adaptation and virulence of their host bacteria, with a high presence in *P. aeruginosa* biofilms (Rice et al., 2009; Secor et al., 2015).

The high-risk clone *P. aeruginosa* ST274 is one of the most prevalent clones in CF patients (Kidd et al., 2012). Genomic analysis shows the presence of complete prophage regions in 3 of the 24 isolates of the *P. aeruginosa* CC274 clone. Inovirus-type phages are present in three different isolates of this clone, two corresponding to previously described phages (Knezevic et al., 2015), and one, the pf8 phage present in the AUS411 isolate, which is a new type of Pf inovirus characterized by the presence of a putative toxin/antitoxin system and a methyltransferase. However, only the AUS531 isolate contains a complete siphovirus type phage, never previously described, the AUS531phi phage.

The Pf8 phage carries a putative novel type of toxin/antitoxin system (Mai-Prochnow et al., 2015), located between an excisionase (acc. no. QGZ15328.1). The genes that encode toxin-antitoxin systems are common in bacteria and are usually located adjacent to genes related to plasmids and other mobile genetic elements (DeShazer, 2004; Dziewit et al., 2007). In prophages preserve their genomes in bacterial hosts via the toxin/antitoxin system, giving them a selective advantage under different stress conditions (Wen et al., 2017).

Temperate bacteriophages can also drive host genome evolution through gene disruption, duplication, transduction or by acting as anchor points for major chromosomal rearrangements (Davies et al., 2016). Previous studies have demonstrated a possible relationship between QS signaling and regulation. The QS system is able to control anti-phage defense mechanisms, leading to lower susceptibility to phage infection in QS-proficient cells. In *Vibrio anguillarum*, QS downregulates expression of the *ompK* gene, thus increasing the resistance to phage KVP40 (Tan et al., 2015; Hoque et al., 2016). In *E. coli*, LamB phage receptors can shield isolates from attack by lytic bacteriophage λ (Høyland-Kroghsbo et al., 2013). However, bacteriophages infect bacteria with a functional QS, as in *P. aeruginosa*, because once the barrier to infection has been overcome it is advantageous for the phage to remain in the genome as a temperate phage improving cooperative behavior by eliminating QS-deficient social cheaters, which not have the phages, despite the fact that phage adsorption is higher in those with QS-deficient strain (Saucedo-Mora et al., 2017). In addition, molecular evolution of clinical strains of *Acinetobacter baumannii* has been demonstrated to have occurred between 2000 and 2010, leading to possession of a functional quorum network and the acquisition of bacteriophages (López et al., 2018).

The QS regulatory role of the *bci* in the prophage was demonstrated by the increase in the expression of this gene in presence of two acyl-homoserine lactone QS inducers, C4-HSL and 3oxo-C12-HSL, which activate the receptors RhlR and LasR, respectively and may induce the *bci* expression (Medina et al., 2003). In the promoter region of the gene, there is a putative *rhl*-*las* box (Subramoni et al., 2015), that have been predicted to be upstream QS-controlled genes (Whiteley et al., 1999). Also, when an infection with the wild prophage AUS531phi and with the mutant phage AUS531phiΔ*bci* were done, the bacterial QS expression was regulated by the wild type phage, which suggest that the *bci* gene has a role in the control of the bacterial QS, favoring the infection by the temperate phages as was also observed in the infection curves.

Virulence factors as pyocyanin production, biofilm and motility are regulated by QS and also influenced by the phage infections (Morkunas et al., 2012; Hosseinidoust et al., 2013; Latino et al., 2014; Castañeda-Tamez et al., 2018; Tariq et al., 2019). The infection with the wild type phage, AUS531phi, carrying the *bci* gene, increased the production of virulence factors, pyocyanin and biofilm, whose presence is characteristic in the lung of CF patients (Castañeda-Tamez et al., 2018). The increase in both biofilm and pyocyanin and a reduction in the swarming motility, are a response to the phage infection which is higher when the *bci* gene is present, but also due to the integration of the temperate phage and the *bci* gene in the bacterial genome, as was described previously. Pyocyanin production has proven to be protective against oxidative stress environments for *P. aeruginosa* (Vinckx et al., 2010). The higher pyocyanin production may be due to a protective response to a higher infectivity capacity of the phage AUS531phi. Temperate phages could help *P. aeruginosa* select for bacterial characteristics that favor persistence of bacteria in the lung (Latino et al., 2014; Tariq et al., 2019). Thus, the *bci* gene may help clinical isolates of *P. aeruginosa* to survive in lung infections, increasing their chance of being infected by temperate phages.

In this research we identified two new prophages, Pf8 and AUS531phi, present in clinical *P. aeruginosa* strains of the CC274 clone, which cause infections in CF patients. Further research is required to determine the role of Pf8 inovirus bacteriophages (filamentous prophages) and their putative toxin/antitoxin system in chronic lung infections by *P. aeruginosa*. Also, we describe a new gene, *bci* (present in prophage AUS531phi), which is involved in regulating the bacterial QS system and favoring the infective capacity of the strain and therefore favoring the presence of this phage in the CF CC274 clone characterized by a low presence of prophages.

## Data Availability Statement

The datasets presented in this study can be found in online repositories. The names of the repository/repositories and accession number(s) can be found below: https://www.ncbi.nlm.nih.gov/genbank/, (https://www.ncbi.nlm.nih.gov/nuccore/MN710383). https://www.ncbi.nlm.nih.gov/genbank/, (https://www.ncbi.nlm.nih.gov/nuccore/MN585195).

## Ethics Statement

This study uses strains obtained from the work titled Clonal dissemination, emergence of mutator lineages and antibiotic resistance evolution in *Pseudomonas aeruginosa* cystic fibrosis chronic lung infection from PlosOne 2013 (PMID: 23951065) and Evolution of the *Pseudomonas aeruginosa* mutational resistome in an international Cystic Fibrosis clone published in Scientific Report 2017 (PMID: 28717172). Research Committee from A Coruña Hospital (Spain) which president is Maria Tomas, confirm it did not require the study to be reviewed or approved by an Ethics Committee because this collection of strains was previously published and in this work is not included clinical data from patients.

## Author Contributions

AA, LB, CL-C, RT, LF-G, IB, MP-A, OP, and ML conducted the experiments, analyzed the results, and wrote the manuscript. RC and TK revised the results. GB and AO revised the manuscript. MT obtained the research funding, directed the experiments, and supervised the writing of the manuscript. All authors contributed to the article and approved the submitted version.

## Conflict of Interest

The authors declare that the research was conducted in the absence of any commercial or financial relationships that could be construed as a potential conflict of interest.

## Footnotes

1 https://www.ebi.ac.uk/~zerbino/velvet/

2 http://www.softberry.com
