# Supplementary material for: Temperate Bacteriophages (Prophages) in Pseudomonas aeruginosa Isolates Belonging to the International Cystic Fibrosis Clone (CC274)
Source: Front Microbiol. 2020 Sep 25;11:556706. doi: 10.3389/fmicb.2020.556706 (PMC7546807; doi:10.3389/fmicb.2020.556706)
Supplement: Supplementary file 1 [file Image_1.pdf]

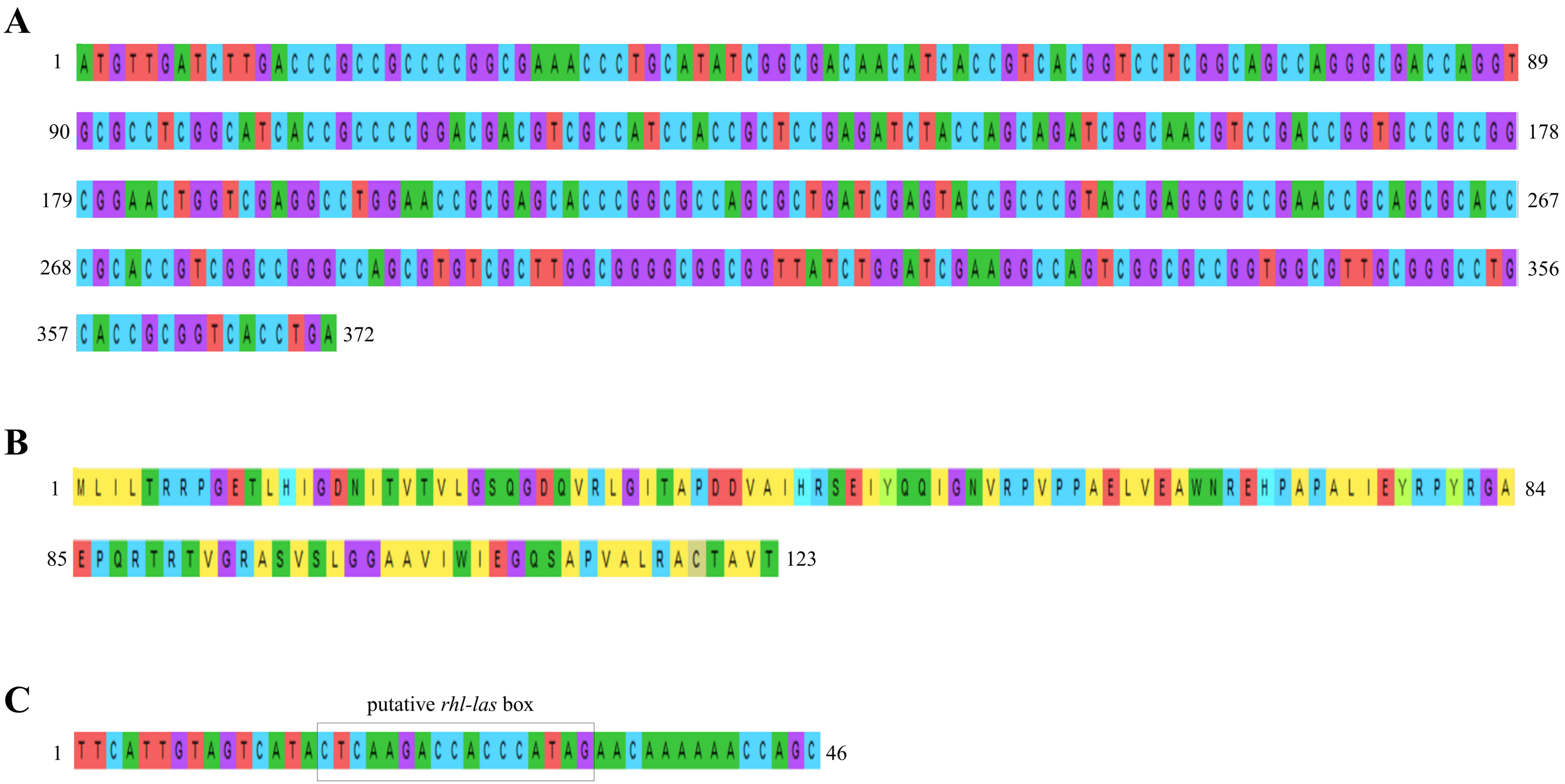

Figure 1S. Graphical representation of the nucleotide sequence of the *bci* gene (**A**), the protein sequence of the *Bci* protein (**B**) and the promoter region of the gene indicating the putative *rhl-las* box (**C**).
